# Iloprost-loaded inhalable Nano into Micro (NiM) formulations for treating hyper-inflammation in a pre-clinical model of cystic fibrosis airway epithelial cells

**DOI:** 10.1007/s13346-026-02101-0

**Published:** 2026-03-21

**Authors:** Annalucia Carbone, Piera Soccio, Emanuela F. Craparo, Pamela Vitullo, Sofia Bonsignore, Pasquale Tondo, Cinzia Scialabba, Namra Sajid, Sobhan Jahani, Salvatore Emanuele Drago, Sante Di Gioia, Donato Lacedonia, Gennara Cavallaro, Massimo Conese

**Affiliations:** 1 Department of Clinical and Experimental Medicine, University of Foggia, Foggia, Italy; 2 Department of Medical and Surgical Sciences, University of Foggia, Foggia, Italy; 3 Department of Biological, Chemical and Pharmaceutical Sciences and Technologies (STEBICEF), University of Palermo, Palermo, Italy; 4 Cystic Fibrosis Support Center, Ospedale “G. Tatarella”, Cerignola, Italy; 5 Institute of Respiratory Diseases, Policlinico Riuniti of Foggia, 71122 Foggia, Italy; 6 Department of Precision Medicine in Medical, Surgical and Critical Areas, University of Palermo, Palermo, Italy

**Keywords:** Cystic fibrosis, Cytokines, MicroRNAs, Iloprost, Polymeric nanoparticles

## Abstract

To address the hyper-inflammation associated with cystic fibrosis (CF) lung disease, we evaluated the efficacy of inhalable Nano into Micro (NiM) formulations carrying a repurposed anti-inflammatory agent, i.e. Iloprost (ILO), a prostacyclin analogue. Nasal epithelial brushings were obtained from 15 individuals with CF bearing at least one *F508del* mutation. The isolated cells were expanded using the conditional reprogramming culture method and subsequently differentiated into a mucociliary epithelium at air–liquid interface (ALI) cultures capable of producing endogenous mucus. ALI cultures were exposed to lipopolysaccharide (LPS), either alone or in combination with NiM formulations – entrapping pegylated or non-pegylated nanoparticles – carrying ILO (named NiM-PEG-ILO and NiM-ILO, respectively). The treatment with NiM-PEG-ILO significantly reduced mRNA levels of the pro-inflammatory cytokines TNF-α, IL-6, IL-8, and IL-1β compared to LPS stimulation alone. Furthermore, a marked downregulation of miR-145, miR-146a, and miR-17 levels was observed relative to the LPS-only group. Cytofluorimetry analysis carried out by using NiM samples entrapping fluorescent pegylated and unpegylated nanoparticles loaded with ILO showed that both nanoparticles NiM formulations were internalized by cells in a concentration-dependent manner. Our results demonstrate that inhalable NiM-based formulations delivery system loaded with ILO can efficiently attenuate inflammation in a pre-clinical model of human airway epithelium.

**Supplementary Information:**

The online version contains supplementary material available at https://doi.org/10.1007/s13346-026-02101-0.

## Introduction

Cystic fibrosis (CF) lung disease begins with an alteration of ion and flux transport across epithelia, due to the lack/dysfunction of the CFTR protein, a chloride and bicarbonate channel. The decrease in chloride transport, along with hyperactivity of sodium epithelial channel (ENaC) brings to a poorly hydrated mucus layer and lack of a proper mucociliary clearance mechanism [1]. Moreover, the acidification of airway surface fluid impairs antibacterial activity [2]. The colonization and the ensuing infection of the airways, favored by these two conditions, are the leading source for a chronic inflammatory state, characterized by heightened secretion of inflammatory mediators, including cytokines [3] and microRNAs (miRNAs) [4]. MiRNAs are implied in the increase of cytokine expression, whereas cytokines, such as TNF-α, IL-1β, and IL-6, activate neutrophils attracted into the CF airways, thus contributing to the tissue damage and chronic inflammation.

CF patients have faced an unprecedented amelioration following the introduction of the CFTR modulator therapy, i.e. the use of small molecule drugs addressing the CFTR protein dysfunctions. The triple therapy, based on two correctors and a potentiatior, i.e. Trikafta (aka Kaftrio), was designed as precision medicine intervention for those bearing at least one *F508del* mutation. Nevertheless, Trikafta has brought major effects on pulmonary functions, such as a meaningful decrease in lung function decline [5]. However, the persistence of chronic bacterial infection [6, 7] and residual airway inflammation [6, 8] in CF patients treated with Trikafta suggests the need for future studies on the role of long-term antibiotic therapy as well as novel anti-inflammatory medications in these patients [9]. CF airway inflammation seems lacking any specific anti-inflammatory drugs that could dampen this dangerous chronic condition. Anti-inflammatory agents, such as steroidal and non-steroidal drugs have not given any significant improvement in clinical outcomes and are plenty of side effects [10].

Prostacyclin analogues (Iloprost, Treprostinil) are approved in the treatment of pulmonary arterial hypertension (PAH) [11–13] due to their potent vasodilator effects, inhibition of platelet aggregation and smooth muscle cell proliferation into the vascular wall, and regulation of innate and adaptive immunity, exhibiting potent anti-inflammatory and immunosuppressive effects [11]. Research has shown that prostacyclin analogues can effectively reduce vascular inflammation by switching off pro-inflammatory transcriptional programs in macrophages and inhibiting the release of cytokines [14, 15]. Iloprost (ILO) has demonstrated prevention of bleomycin-induced pulmonary fibrosis in mice, possibly by downregulating pro-inflammatory and pro-fibrotic cytokines [16]. Additionally, prostacyclin analogues, by increasing cAMP levels, successfully enhance the expression and gating of the F508del-CFTR protein [17]. Therefore, prostacyclin analogues emerge as excellent candidates for repositioning as inhalable drugs for the treatment of hyper-inflammation of lung tissue associated with CF.

Aerosolized dry powder formulations present an appealing approach for lung drug delivery, enhancing therapeutic efficacy at the target site while minimizing systemic side effects [18, 19]. Formulations must be meticulously designed to facilitate proper deposition in the bronchial tree and overcome the challenging traversal of the mucus gel, known for its size and interaction filtering properties. To address this, Nano into Micro (NiM) particles have been developed, combining a micrometric matrix for effective aerosolization and bronchial tree deposition with nanometric carriers (loaded with ILO) designed to penetrate the thick mucus layer in CF patients and modulate the drug’s in vivo fate upon administration [20, 21]. We have recently shown that the ILO-loaded polymeric nanoparticles embedded into NiM particles exhibited high cytocompatibility and a pronounced anti-inflammatory effect toward CFBE cells overexpressing F508del CFTR (CFBE-F508del) in terms of reduction of IL-1β, TNF-α, IL-6, and IL-8 gene expression [20]. The aim of this work was to assess their efficacy and uptake in a pre-clinical model of ex-vivo CF differentiated airway epithelial cells using as biomarkers cytokines and microRNAs (miRNAs). The hyper-inflammatory state associated with CF is still an orphan drug condition. To tackle this drawback and to align with precision medicine principles, the cohort of patients considered within this study was highly homogeneous, i.e. they were homozygotes or heterozygotes for the most frequent mutation in Western world, that is *F508del*, and were treated with the triple therapy Trikafta. We established a human cell culture model that closely mimics the in vivo airway epithelium and that has been instrumental for assessing and optimizing the efficacy of the CFTR modulators, including Trikafta, before applying them to the patients [22]. Indeed, human nasal epithelial cells (hNECs) are endowed with morphological, electrophysiological and transcriptomic features that reflect several characteristics of the conducting airways [23, 24]. Furthermore, hNECs are considered a good surrogate for the bronchial cells, with the advantage of being obtained with less invasiveness and, nevertheless useful for analyzing CFTR currents [23].

## Materials and methods

### Synthesis and characterization of PHEA-*g*-PLGA and PHEA-*g*-PLGA-*g*-PEG, PHEA-*g*-FITC-*g*-PLGA and PHEA-*g*-FITC-*g*-PLGA-*g*-PEG graft copolymers

FITC (0.02 mmol) was dissolved in anhydrous DMF (1 mL), PHEA (1.71 mmol repeated units (RU)) was simultaneously dissolved in anhydrous DMF (6 mL); after complete solubilization, FITC was added dropwise to the PHEA dispersion. The amounts were calculated according to R_1_ = mmol FITC/mmol PHEA RU = 0.0045. The resulting reaction mixture was left under stirring at 40 ± 0.1 °C for 16 h. Residual organic solvents remain after the washing steps were removed under vacuum. The solid was then dissolved in double-distilled water and further purified by gel filtration chromatography, using Sephadex G25 medium resin as the stationary phase (particle size between 50–150 μm) and double-distilled water as the mobile phase. At the end of the purification process, the fraction containing the copolymer was lyophilized. The PHEA-*g*-FITC copolymer was obtained with a yield of 80 wt%. Functionalization was evaluated by UV analysis, comparing the obtained absorbance at 517 nm of the aqueous copolymer dispersion with a calibration curve made by FITC alone; it was found equal to 0.44 mol%.

Functionalization of PHEA or PHEA-*g*-FITC with PLGA was achieved using 1,1’-carbonyldiimidazole (CDI) as a coupling agent [20]. PLGA (0.085 mmol) was dissolved in anhydrous DMF (10 mL); after complete solubilization, CDI (27.7 mg; 0.17 mmol) was added. The solution was stirred at 40 °C for 4 h. At the same time, PHEA or PHEA-*g*-FITC (225 mg; 1.42 mmol RU) was dissolved in anhydrous DMF (6 mL); diethylamine (DEA) (0.425 mmol) was added to the PHEA-*g*-FITC dispersion about 15 min before it was added to the activated PLGA dispersion. After this time, the latter dispersion was added dropwise to the activated PLGA dispersion. The resulting reaction mixture was left under stirring at 40 ± 0.1 °C for 48 h. The amounts of each reagent were properly determined accordingly to R_2_ = (mmol of PLGA/mmol of PHEA or PHEA-*g*-FITC RU) = 0.06, R_3_ = (mmol of CDI/mmol of PLGA) = 2 and R_4_ = (mmol of DEA/mmol of PLGA) = 5. After this time, the copolymer was precipitated in an appropriate amount of ethyl ether, separated by centrifugation at 10,000 rpm, at 4 °C for 10 min, washed two times with a dicloromethane/ethyl ether mixture (ratio 55:45 v/v). Then, the obtained product was dried under vacuum. PHEA-*g*-PLGA and PHEA-*g-*FITC-*g*-PLGA graft copolymers were obtained with a yield of 70 wt % based on the starting amounts of PHEA and PLGA.

^1^H NMR spectra were registered by a Bruker Advance II-300 spectrometer working at 300 MHz (Bruker, Milan, Italy).

PHEA-*g*-FITC-*g*-PLGA ^1^H-NMR (300 MHz, DMF-d_7_, 25 °C, TMS): *δ* 1.5–1.9 ppm (m, 3-H_PLGA_ –[OCOCH(C**H**_**3**_)]_92_–); *δ* 3.0 ppm (m, 2H_PHEA_ –COCHC**H**_**2**_CONH–); *δ* 3.5 ppm (t, 2H_PHEA_ –NHC**H**_**2**_CH_2_O–); *δ* 3.6 ppm (t, 2H_PHEA_ –NHCH_2_C**H**_**2**_O–); *δ* 5.0 ppm (m, 1H_PHEA_ –NHC**H**(CO)CH_2_–); *δ* 4.2–4.5 and 5.4–5.8 ppm (1H_PLGA_, –[OCOC**H**(CH_3_)]_92_–); *δ* 5.3 ppm (2H_PLGA_, –[OCOC**H**_**2**_]_92_–). Derivatization degree in PLGA (DD_PLGA_) is equal to 3.5 ± 0.2 mol%.

Functionalization of PHEA-*g*-PLGA or PHEA-*g*-FITC-*g*-PLGA with H_2_N-PEG (Mw = 2,000 Da) by using triethylamine (TEA) and N,N′-disuccinimidyl carbonate (DSC) was achieved by following an already published procedure [20]. Briefly, PHEA-*g*-PLGA or PHEA-*g*-FITC-*g*-PLGA (0.52 mmol PHEA RU) was dissolved in anhydrous DMA (6 mL); after complete solubilization, TEA (7,41 l; 0,052 mmol) and DSC (13,5 mg; 0,052 mmol) were added. The solution was stirred at 40 °C for 4 h. At the same time, H_2_N-PEG (79 mg; 0,039 mmol) was dissolved in anhydrous DMA (5 mL). After the activation time the activated PHEA-*g*-PLGA or PHEA-*g*-FITC-*g*-PLGA dispersion was added dropwise to the activated H_2_N-PEG dispersion. The resulting reaction mixture was left under stirring at 25 ± 0.1 °C for 18 h. The amounts of each reagent were properly determined according to R_5_ = (mmol H_2_N-PEG/mmol PHEA RU) = 0.075, R_6_ = (mmol of DSC/mmol of PHEA RU) = 0.1 and R_7_ = (mmol TEA/mmol DSC) = 1.

After this time, the solution was purified by dialysis (RC Turbing, at MWCO 25 kDa), against bidistilled water, subsequently, the solution was freeze-dried and stored for further characterization. The PHEA-*g*-PLGA and PHEA-*g*-FITC-*g*-PLGA copolymers were obtained with a yield of 80 wt% based on the starting amounts of PHEA-*g*-PLGA or PHEA-*g*-FITC-*g*-PLGA and H_2_N-PEG.

PHEA-*g*-FITC-*g*-PLGA-PEG ^1^H-NMR (300 MHz, *d*_7_-DMF, 25 °C, TMS): δ 1.5–1.9 (m, 3H_PLGA_ − [OCOCH(C**H**_**3**_)]_92_ −); δ 2.8 (m, 2H_PHEA_ − C(O)CHC**H**_**2**_C(O)NH −); δ 3.5 (t, 2H_PHEA_ − NHC**H**_**2**_CH_2_O −); δ 3.6 (t, 2H_PHEA_ − NHCH_2_C**H**_**2**_O −); δ 3,7 (4H_PEG_, − [C**H**_**2**_C**H**_**2**_O]_44_ −); δ 5.0 (m, 1H_PHEA_ − NHC**H**(CO)CH_2_ −); δ 4.2–4.5 and δ 5.4–5.8 (1H_PLGA_ − [OCOC**H**(CH_3_)]_92_ −); δ 5.3 (2H_PLGA_ − [OCOC**H**_**2**_]_92_ −). Derivatization degree in PEG (DD_PEG_) is equal to 4.2 ± 0.2 mol%.

### Preparation and characterization of Iloprost-Loaded Nanoparticles

ILO-loaded nanoparticles, pegylated or unpegylated, fluorescent or unlabelled, were obtained respectively, starting from the PHEA-*g*-PLGA-*g*-PEG, PHEA-*g*-PLGA, and PHEA-*g*-FITC-*g*-PLGA-*g*-PEG and PHEA-*g*-FITC-*g*-PLGA graft copolymers by following procedure reported elsewhere [20]. Samples were named respectively NP-PEG@ILO, NP@ILO, FNP-PEG@ILO and FNP@ILO. The mean size (nanometers) and polydispersity index (PDI) ζ potential measurements of obtained nanoparticles were determined by a Malvern Zetasizer NanoZS (Malvern Instruments, Worcestershire, UK) as already reported [20]. Each sample was dispersed in ultrapure water and analyzed at 25 °C in triplicate. The content of ILO loaded into the nanoparticle samples, expressed as Drug Loading (DL%), was determined by high-performance liquid chromatography (HPLC) analysis as reported elsewhere [20].

### Preparation and characterization of NiM formulations

Nano into micro (NiM) formulations were prepared by using a Mini Spray Dryer B-290 (Buchi, Milan, Italy) and the experimental procedure already reported [20]. Starting from a liquid feed composition containing NP-PEG@ILO, NP@ILO, FNP-PEG@ILO and FNP@ILO, NiM samples named respectively NiM-PEG-ILO, NiM-ILO, NiM-fluo-PEG-ILO and NiM-fluo-ILO were obtained. The content of ILO loaded into NiM samples was determined by high-performance liquid chromatography (HPLC) analysis as reported elsewhere [20]. The DL% for all the samples was found equal to 0.023 ± 0.004 wt%.

The aerodynamic behavior of samples NiM-PEG-ILO and NiM-ILO (obtained by adding rhodamine at 0.04% wt/vol as fluorescent tracer) was assessed using a Handihaler® dry powder inhaler (DPI) connected to an Andersen Cascade 8 Stage Impactor (ACI, InPharmaTec) operating at an airflow of 90 l/min. To improve particle deposition during aerosolization, the impaction cups of all ACI stages were coated with 1% (v/v) Tween 80 in ethanol and dried before testing. Two methylcellulose capsules (size 3) were filled with 40 mg of powder and aerosolized for 5 s using a Bravo X Bio pump. After deposition, powders were recovered and quantified spectrophotometrically at 540 nm using aqueous microparticle dispersions as standards. Aerodynamic performance was assessed from the ACI deposition profile by calculating the Mass Median Aerodynamic Diameter (MMAD), Geometric Standard Deviation (GSD), and Fine Particle Fraction (FPF%), the latter representing the fraction of particles < 5 μm and indicating their potential for deep lung deposition.

Flowability of NiM particles was expressed as the Carr index (calculated using the following formula):

$$Carr\;Index=(\rho_{tapp}-\rho_{bulk})/\rho_{tapp}\times100$$

were ρ_tapp_ is the tapped density, and ρ_bulk_ is the bulk density, calculated as reported elsewhere [21].

### Study participants and ethics statement

CF patients (> 18 years) with *F508del*/*F508del* and *F508del*/other mutation genotypes were included in this study. Patients were enrolled at Cystic Fibrosis Support Center of the “G. Tatarella" Hospital (Cerignola, Italy), had a confirmed CF diagnosis based on sweat chloride testing and/or genotyping, and were on CFTR modulator therapy (Trikafta) for at least 15 days prior to sample acquisition. The studies described in this article were approved by the Ethics Committee of the Riuniti Hospital of Foggia (no. 124/CE/2023, issued on 26/07/2023) and were conducted in compliance with the current Good Clinical Practice standards and in accordance with the relevant guidelines and regulations and the principles set forth under the Declaration of Helsinki (1989). During this study, the privacy rights of participants were observed and informed consent was obtained.

### Collection and processing of primary cells

Primary human nasal epithelial cells (hNECs) were obtained through cytology brushing of the nasal inferior turbinate of CF participants. hNECs were dislodged from cytology brushes by gentle vortexing. Cells were pelleted at 300 g for 5 min at 4 °C and resuspended in the expansion culture media [DMEM/Ham’s F-12 (Merck, Cat# D8437) and DMEM high glucose (Merck, Cat# D6429) at a 2:1 ratio] supplemented with 5% Fetal Bovine Serum (FBS, Corning, Cat# 35–010-CV), 1% penicillin and streptomycin (Corning, Cat# 30–002-Cl), 25 µg/mL amphotericin B (Corning, Cat# 30–003-CF), insulin 5 μg/mL (Merck, Cat# I2643), hydrocortisone 0.4 μg/mL (Merck, Cat# H0888), cholera toxin 8.4 ng/mL (Merck, Cat# C8052), human Epidermal Growth Factor (hEGF) 25 ng/mL (Merck, Cat# E9644), adenine 24 μg/mL (Merck, Cat# A2786), and ROCKi Y-27632 10 μM (Selleck Chemicals, Cat# S1049). Then, the resuspended cells were passed through a 40 µm cell strainer (Merck, Cat# CLS431750), to generate single-cell suspension, and seeded into flasks for serial expansion using conditionally reprogrammed cell culture.

### Conditionally reprogrammed cell (CRC) expansion culture method

hNECs were added to collagen I-coated flask and grown in expansion culture media containing the ROCKi Y-27632. Media change was performed every second day until 80–90% confluence. Cells were dissociated using accutase solution (Merck, Cat# A6964) and cell count was performed using Countess II automated cell counter (Thermo Fisher Scientific, Waltham, MA) according to the manufacturer’s instructions. Cell viability at each passage exceeded 93% in average. hNECs used for the MTT assay (*n* = 3), or seeded for ALI cultures and then analysed for gene expression (*n* = 10), for uptake (*n* = 4) or for immunofluorescence and confocal microscopy (*n* = 2). Cell doubling time was calculated as (72*ln(2*)*/*ln*(final number of cells/initial number of cells)).

### Cytobiocompatibility of NiMs with hNECs

Cellular cytotoxicity of NiMs were assessed using the MTT (3-(4,5-dimethylthiazol-2-yl)−2,5 diphenyl tetrazolium bromide) assay [25]. 96-well plates were used to seed the hNECs cells at a seeding density of 5 × 10^3^ cells/well. Starting from lyophilized samples, NiMs were dissolved in the expansion culture medium (see above) overnight under agitation. After 72 h, serial dilutions of NiM-ILO and NiM-PEG-ILO were added and allowed to incubate for 4 and 24 h at 37 °C under 5% CO_2_. MTT solution (0.5 mg/mL) was added after the removal of the medium and left to incubate for a further 3 h. At the end of the incubation, the MTT solution was removed carefully, and the formed purple formazan crystals were dissolved in DMSO (dimethyl sulfoxide). The absorbance of the dissolved purple formazan was measured at 595 nm. The cell viability was calculated as follows: % viability = [(Optical density {OD} of treated cell – OD of blank)/(OD of vehicle control – OD of blank) × 100], considering untreated cells as 100% and cells treated with 1% Triton X-100 were used as positive control. IC50 (half-maximal inhibitory concentration) values were calculated, considering incubation hNECs with eight different NiM concentrations (3.5, 35, 350, 1750, 3500, 5250, 7000, and 10,500 nM) at 24 h, by linear regression. Two experiments were performed with a total of six replicates.

### Air–liquid interface (ALI) cultures

Passage two hNECs CRC-expanded cells were seeded on Transwell 6.5 mm, 0.4 µm pore polyester membrane inserts (0.33cm^2^ area; Merck, Cat# CLS3470) pre-coated with collagen I (Corning, Cat# 354,236), at a density of 150,000 cells/insert until confluence. Once confluent under submerged conditions (approximately 4–5 days), cells were switched to air–liquid interface (ALI) culture condition, whereby apical media was removed, and PneumaCult ALI differentiation media (STEMCELL Technologies, Cat# 05001), without hydrocortisone, was added to the basolateral compartment only. Basal media change was performed every second day for 3–4 weeks. The apical surface was washed with warm phosphate buffered saline (PBS) 1X (Corning, Cat# 20–031-CV) once a week to remove excess mucus.

### Trans-epithelial electrical resistance (TEER) meaures

To assess differentiation in primary hNECs, measurement of TEER was performed using chopstick electrodes coupled with an ohmmeter (Millicell-ERS, Millipore, Billerica, Massachusetts, USA), after the addition of 200 µL medium to the apical surface of cultures. Epithelial differentiation was achieved for TEER values of at least 400–500 Ω/cm^2^.

### Immunofluorescence and confocal microscopy

Immunofluorescence and confocal miscoscopy were performed as previously published [26]. Cells on transwell membranes were fixed in 4% paraformaldehyde (Carlo Erba Reagents, Cat# 415,694) for 20 min at room temperature, and then were washed with PBS at room temperature for three times; after fixation, cells were permeabilized with 0.1% Triton-X (Fluka BioChemika, Cat# 93,418) in PBS for 5 min. After washed three times with PBS at room temperature, fixed and permeabilized hNECs were blocked using 5% BSA (Merck, Cat# A9418) in PBS for 2 h at room temperature. Cells were then incubated for 2 h at room temperature with Alexa Fluor™ 488 mouse ZO-1 monoclonal antibody (Thermo Fisher Scientific, Cat# 33–9111), FITC-conjugated mouse anti-α-tubulin monoclonal antibody (Merck, Cat# F2168), or Alexa Fluor® 488 mouse anti-mucin 5AC monoclonal antibody (abcam, Cat# ab309610); all antibodies were diluted 1:50 with 1% BSA and 0.1% Tween 20 (Merck, Cat# P7949) in PBS. Cells were rinsed three times with PBS, then filters were excised and placed side up on a glass slide, and overlayed with a drop of VECTASHIELD® Antifade Mounting Medium containing DAPI (Vector Laboratories, Cat# H-1200) followed by a coverslip. Images were acquired using a Nikon Eclipse Ti-E C2 confocal microscope (Nikon Europe B.V., Amstelveen, The Netherlands). Specimens were viewed through a 40 × objective. Digital images were acquired in the horizontal (x–y) and in the vertical (x–z) planes by using the program NIS Element (Nikon) and processed using ImageJ software (National Institute of Health, Bethesda, MD).

### In silico analysis

Bioinformatic analysis was performed using the miRNA target prediction databases TargetScanHuman 8.0 (https://www.targetscan.org/vert_80/) and miRTarBase (https://mirtarbase.cuhk.edu.cn/~miRTarBase/miRTarBase_2025/php/index.php) to search for miRNAs predicted to target the 3′UTR sequence of IL-1β, TNF-α, IL-6 and IL-8. The following associations were found: miR-17 with IL-8; miR-146a with IL-6 and TNF-α; miR-145 with IL-1β.

### RNA extraction from Iloprost- and NiM-treated hNECs

Free iloprost and NiMs were dissolved in PneumaCult ALI differentiation medium overnight under agitation and were used for treatment by adding them to the apical compartment. In particular, differentiated hNECs were co-treated for 4 h at 37 °C with ALI differentiation media containing 10 µg/ml LPS from *P. aeruginosa* (Merck, Cat# L8643) ± 350 nM free Iloprost, or 350 nM of NiM-ILO or 350 nM of NiM-PEG-ILO. Then cells were lysed and total RNA was extracted using TRIzol™ Reagent (Thermo Fisher Scientific, Cat# 15,596,018) in combination with the Total RNA Purification Kit (Norgen Biotek, Cat# SKU 17200), following the manufacturer’s protocol. RNA concentration and quality was measured using the NanoDrop ONEc spectrophotometer (Thermo Fisher Scientific, Waltham, MA), while purity was assessed by the OD260/OD280 ratio.

### RNA reverse transcription and expression of miRNA through q-PCR

2 ng of total RNA was used for cDNA synthesis using the TaqMan MicroRNA RT Kit (Thermo Fisher Scientific, Cat# 4,366,596), according to the manufacturer’s protocol. Quantitative real-time polymerase chain reaction (qRT-PCR) assays for the quantitative determination of miRNA expression were performed in duplicate using the ABI-PRISM 7300 (PE Applied Biosystems, Waltham, MA, USA). U6 snRNA was used as an endogenous control. miRNA expression was calculated using the comparative 2^−∆∆CT^ method. Table 1 reports the miRNA sequences analysed in this study.

**Table 1:** miRNAs sequences

Assay Name	Assay ID	Species	Mature miRNA Sequence
U6 snRNA	001973	Human	GTGCTCGCTTCGGCAGCACATATACTAAAATTGGAACGATACAGAGAAGATTAGCATGGCCCCTGCGCAAGGATGACACGCAAATTCGTGAAGCGTTCCATATTTT
hsa-miR-17-5p	002308	Human	CAAAGUGCUUACAGUGCAGGUAG
hsa-miR-146a-5p	000468	Human	UGAGAACUGAAUUCCAUGGGUU
hsa-miR-145-5p	002278	Human	GUCCAGUUUUCCCAGGAAUCCCU

### Gene expression of inflammatory cytokines

Concurrently, total RNA was reverse transcribed into cDNA using the High Capacity cDNA Reverse Transcription Kit (Thermo Fisher Scientific, Cat# 4,368,814), following the manufacturer’s protocol. The expression of Interleukin 6 (IL-6, assay ID Hs00174131_m1), Interleukin-1 Beta (IL-1β, assay ID Hs01555410_m1), Interleukin 8 (CXCL-8, assay ID Hs00174103_m1) and Tumor Necrosis Factor (TNF-α, assay ID Hs00174128_m1) genes, all from Thermo Fisher Scientific, was then assessed by qRT-PCR with TaqMan™ Universal Master Mix II, no UNG (Thermo Fisher Scientific, Cat# 4,440,047), as specified by the manufacturer. Real-time PCR reactions were performed in duplicate for each sample using 96-well plates and a reaction volume of 20 µL, containing 1X TaqMan™ Universal Master Mix II, no UNG, 1X TaqMan® Gene Expression Assay, and 100 ng of cDNA, with β-actin (Actb, assay ID Hs01060665_g1) from Thermo Fisher Scientific used as an endogenous control. The reactions were conducted on the ABI-PRISM 7300 instrument (PE Applied Biosystems, Waltham, MA, USA). Relative quantification was performed using the comparative 2^−ΔΔCT^ method.

### Uptake assay

To evaluate the efficiency of uptake of NiM by cells, after differentiation hNECs were incubated with NiM carrying fluorescein isothiocyanate (FITC) at the final concentration of 350, 1900 and 3500 nM ILO. After 4 h of incubation at 37 °C, cells were treated with trypan blue (0.05% in PBS) to quench extracellular fluorescence [27], detached, resuspended in 500 µl PBS 1X and then analyzed by Amnis Flowsight IS100. Brightfield and green fluorescent images for any single cell event were collected. In another set of experiments, cells were analysed by Attune™ NxT Acoustic Focusing Flow Cytometer. Brightfield scatter plots obtained by plotting area on x-axis vs aspect ratio on y-axis were generated, then single cells events were gated, and finally 10,000 single-cell events for sample were acquired. The percentage of green positive cells (channel 2, 488 nm excitation laser) and mean fluorescence intensity were analyzed using Attune NxT 3.2 Software.

### Statistical analysis

Comparisons between groups were performed by ANOVA or Kruskal–Wallis test depending on whether the data were normally distributed or not. The data are presented as mean ± standard deviations (SD), mean ± standard error of the means, or medians and interquartile range. A p-value < 0.05 has been considered statistically significant. Due to the low number of samples (*n* = 10), we computed the Spearman rank correlation coefficients (r_s_) between parameters. GraphPad Software (version 9.0, GraphPad Software) was used for the analysis.

## Results

### Preparation and characterization of Nano into Micro (NiM) particles

Nano into micro (NiM) particles were developed as previously reported [20]. In particular, iloprost (ILO)-loaded polymeric pegylated nanocarriers (named NP-PEG@ILO) with negatively charged, hydrophilic surfaces and reduced interactions with mucins, were produced as already reported [21]. In detail, the starting polymeric material to produce nanoparticles was obtained from the synthetic polyamino acid α,β-poly(N-2-hydroxyethyl)-D,L-aspartamide (PHEA), which was subsequently functionalised with poly(lactic-*co*-glycolic acid) (PLGA), obtaining the PHEA-*g*-PLGA graft copolymer, and with poly-(ethylene glycol) (PEG) to obtaining the PHEA-*g*-PLGA-*g*-PEG graft copolymer. As control sample, ILO-loaded unpegylated nanoparticles (named NP@ILO) were obtained by using as starting material the PHEA-*g*-PLGA graft copolymer. The chemical physical properties of the obtained samples in terms of Z-average, PDI, ζ potential values and Drug Loading (DL%, the weight ratio between the entrapped drug and the total sample) are reported in Table 2.

**Table 2:** Z-average, PDI, ζ potential values, and DL% of all obtained samples

Sample	Z-average (nm)	PDI	ζ Potential (mV)	DL% (wt%)
NP@Ilo	142.50	0.321	−38.7	0.15
NP-PEG@ILO	141.42	0.273	−29.5	0.13
FNP@ILO	158.10	0.312	−32.3	0.15
FNP-PEG@ILO	159.38	0.305	−19.2	0.14

**Fig. 1:**
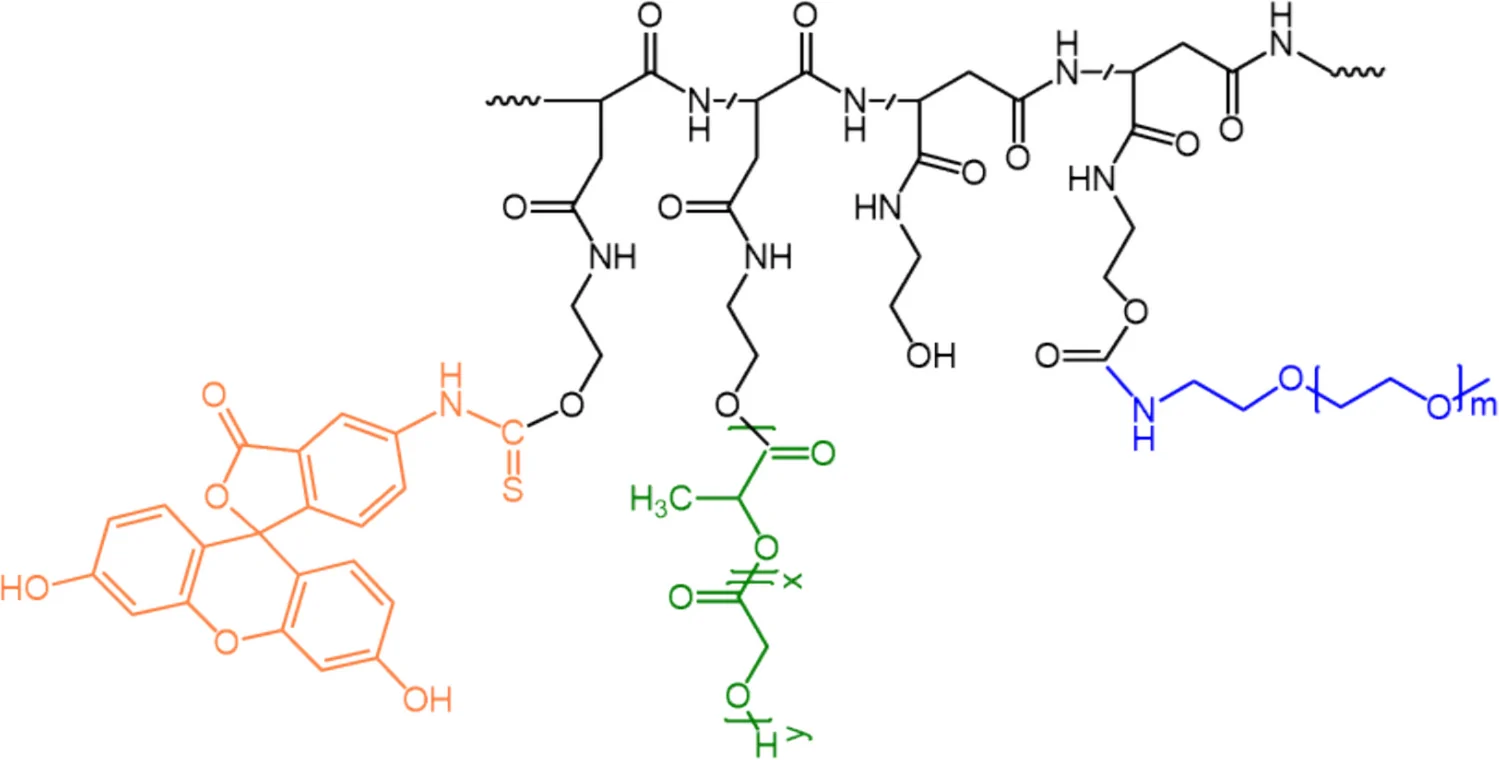
Chemical structure of PHEA-*g*-FITC-*g*-PLGA-*g*-PEG graft copolymer (x and y = 92; m = 44)

As can be seen, the nanoparticle samples show a Z-average lower than 200 nm (that is essential to achieve the nanoparticle mucodiffusion), negative ζ potential and similar drug loading (DL%). The PDI values are slightly high but consistent with the nanoparticle preparation method based on nanoprecipitation and the absence of additional excipients that could improve size uniformity. In any case, the mean diameter values, expressed as Z-average, are all below 160 nm. Therefore, the observed polydispersity still indicates particles within a consistently small size range. FITC-conjugated polymeric nanoparticles, pegylated or unpegylated (named FNP-PEG@ILO and FNP@ILO), were obtained respectively from the PHEA-*g*-FITC-*g*-PLGA-*g*-PEG and the PHEA-*g*-FITC-*g*-PLGA graft copolymers. The chemical structure of the PHEA-*g*-FITC-*g*-PLGA-*g*-PEG is represented in Fig. 1.

The chemical physical properties of both FNP-PEG@ILO and FNP@ILO samples, also reported in Table 2, showed that fluorescent samples have no significant differences than the not labelled ones, with comparable DL%, indicating that the FITC covalently linked to the polymeric backbone does not influence the nanoprecipitation of the graft copolymers.

To achieve an inhalable formulation, the NiM strategy was employed by spray-drying (SD), using as liquid feed an aqueous dispersion of polymeric nanoparticles in a mannitol-containing solution [20, 21]. Mannitol, a clinically used osmotic agent, is able to increase water flux into the bronchial lumen and improve mucus clearance in CF patients [28–31]. By using the dispersion of pegylated or unpegylated nanoparticles to be used as control, two NiM samples were produced, which were named respectively NiM-PEG-ILO and NiM-ILO. NiM particles were also produced starting from pegylated or unpegylated FITC-conjugated polymeric nanoparticles (FNP-PEG@ILO and FNP@ILO samples, respectively), following the same procedure reported before for unlabelled NiM formulations, which were named NiM-fluo-PEG-ILO or NiM-fluo-ILO. Due to the presence of a microparticle matrix made by mannitol, these NiM samples quickly dissolve once in contact with the aqueous fluids, rapidly lose their structural integrity upon contact with a physiological fluid (such as cell culture medium), releasing the iloprost-loaded nanoparticles, as already reported [20], which chemical-physical characteristics are comparable to those reported in Table 2 for fresh nanoparticle dispersions (data not shown).

**Fig. 2:**
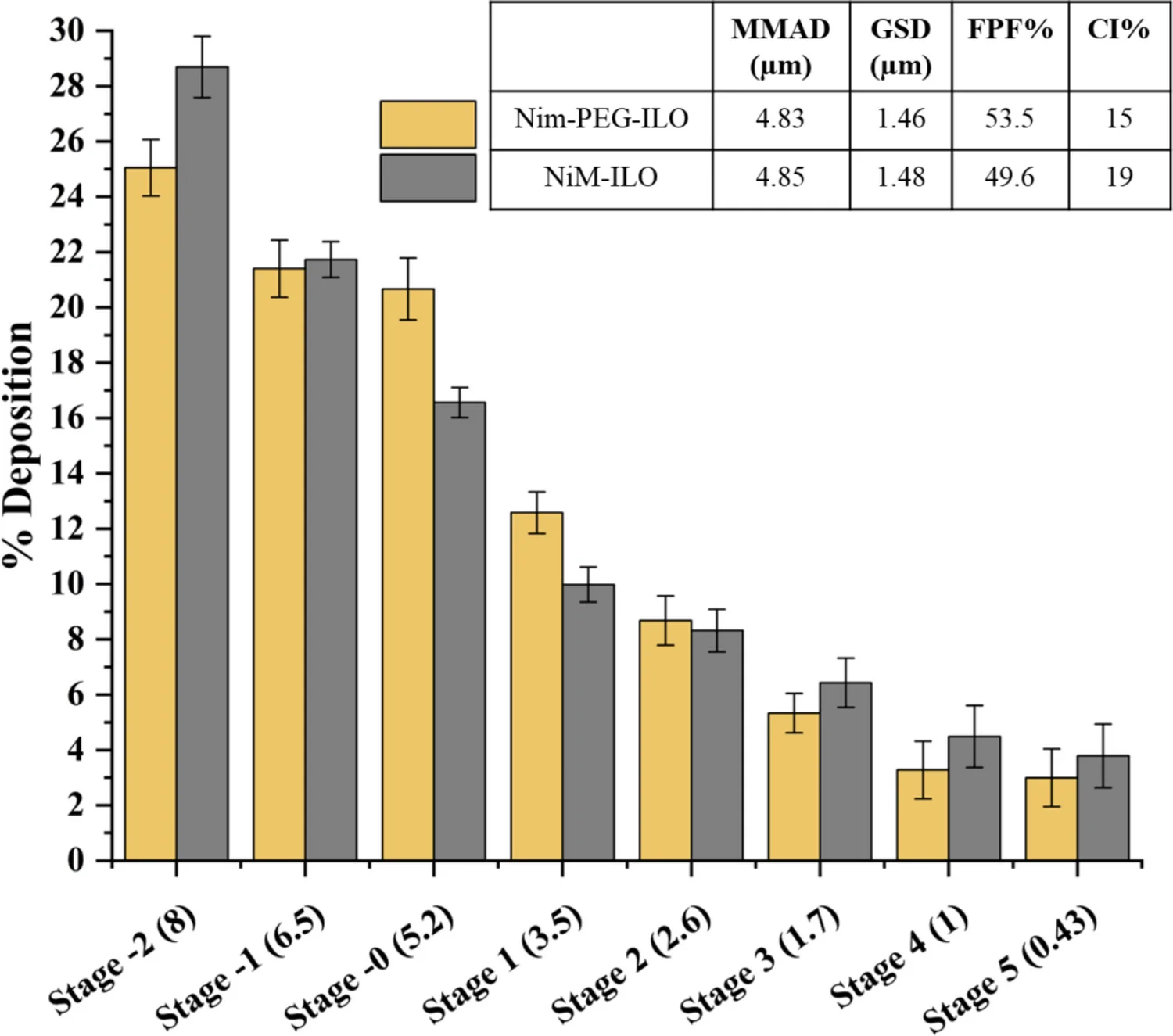
Deposition profile and aerodynamic parameters of NiM-PEG-ILO and NiM-ILO samples, after testing via Handihaler operating at a rate airflow of 90 l/min. Data are expressed as means ± SD (*n* = 3)

To explore the suitability of the obtained microparticle-based powders for pulmonary administration, the flow properties and the aerodynamic performance of the NiM samples were assessed. A good flowability, expressed as Carr index (CI) (see experimental part), was found for both NiM-PEG-ILO and NiM-ILO, being the CI equal to 15 and 19, respectively. This result reflects a slight difference between the bulk density (ρ_bulk_) and the tapped density (ρ_tapp_) for both samples, with better flowability for the NiM-PEG-ILO sample. Moreover, aerosolization of both NiM-PEG-ILO and NiM-ILO samples through a standard Handihaler® dry powder inhaler (DPI) connected to an Andersen Cascade Impactor (ACI) showed also good aerodynamic properties, as illustrated in Fig. 2, being the the aerodynamic parameters, mass median aerodynamic diameter (MMAD), Geometric Standard Deviation (GSD), and fine particle fraction (FPF, defined as particles under 5 μm in diameter) resulted equal to 4.83 μm, 1.46 and 53.5% for NiM-PEG-ILO sample; these results were comparable to those obtained with NiM-ILO. Therefore, these findings support the potential use of these NiM powders for inhalation ad dry powder, promoting efficient deposition within the lower respiratory tract.

### Patients’ demographics and clinical status

The patients (*n* = 15) selected were all with *F508del* mutation in homozygosity or heterozygosity, with age > 18 years and with a body mass index (BMI) denoting a normal weight status. Although age at diagnosis was very early, initiation of Trikafta therapy was started at a median age of 23 years. Respiratory function (FEV_1_) was normal. The subjects had respiratory infections from bacteria that are typical of this disease, namely *P. aeruginosa*, *Burkholederia cepacia*, and *Staphylococcus aureus*. Hematological parameters did not denote significant alterations from normal values (Table 3). Although mucus expectoration was lowered and clinical symptoms were relieved by triple therapy, patients were still colonized by pathogens, denoting that the triple therapy did not resolve all patients’ pathological conditions.

**Table 3:** Demographic and clinical characteristics of CF patients (*n* = 15)

Section	Parameter	Value
Anthropometric Data		
	Sex (M/F)	9/6
	Age (years)	38 (23–50)
	BMI (kg/m^2^)	22.5 (21.6–26.7)
Genotype		
	*F508del*/*F508del*	3
	*F508del*/other mutation	12
Clinical Data		
	Age at diagnosis (years)	0.6 (0.2–5.0)
	Age of starting triple therapy (years)	34 (22–45)
	FEV_1_% predicted	81 (73–106)
Respiratory Infections		
	*Pseudomonas aeruginosa*	4
	*Staphylococcus aureus*	4
	*Burkholderia cepacia*	3
	MRSA	1
	*Haemophilus influenzae*	1
	*Achromobacter xylosoxidans*	1
	Not detected	1
Haematological Parameters		
	C-reactive protein (mg/L)	0.09 (0.04–0.43)
	Total WBC (n/mm^3^)	5460 (4800–8440)
	Neutrophils (%)	57.4 (52.0–68.0)
	Lymphocytes (%)	33.0 (20.4–37.0)
	Monocytes (%)	7.3 (4.1–9.4)
	Basophils (%)	0.4 (0.3–0.7)
	Eosinophils (%)	3.4 (1.5–4.0)

*BMI*: body mass index; *FEV*_1_: forced expiratory volume in 1 second; *MRSA*: Methicillin-Resistant *Staphylococcus aureus. *Data are reported as medians (interquartile range)

### Cytobiocompatibility of NiM formulations with primary hNECs

**Fig. 3:**
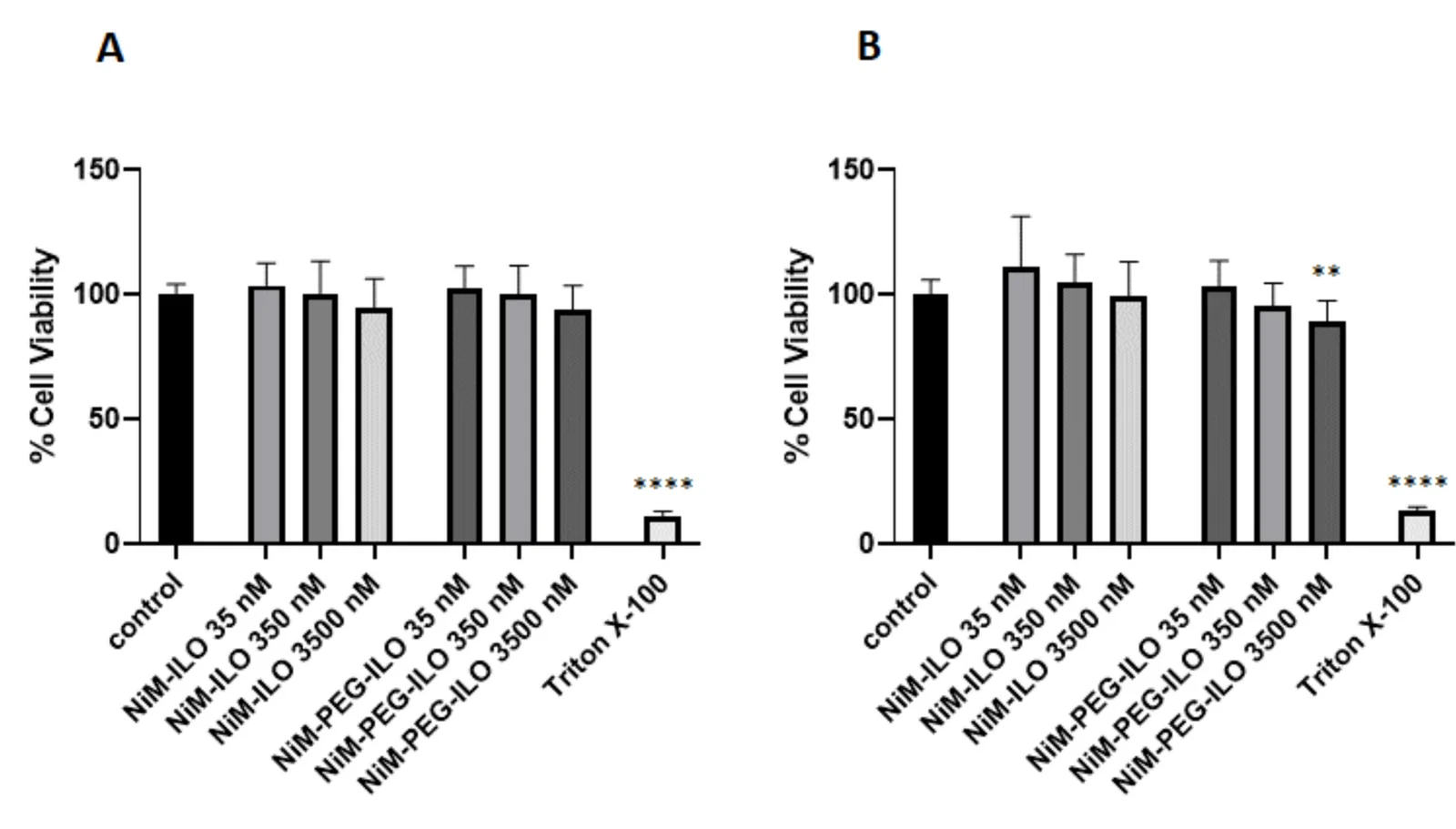
Cytotoxicity of NiM formulations. hNECs from three patients were challenged for 4 h (**A**) or 24 h (**B**) with NiM-ILO Ilo and NiM-PEG-ILO at the indicated concentrations of ILO (in nM). Cells were then assayed for vitality by the MTT assay. Untreated cells (control) represented 100% viability, whereas 1% Triton X-100 was used as positive control. Data represent the mean ± SD of eight replicates for each condition and per each patient (thus having 24 replicates for each condition). **p < 0.01; ****p < 0.0001 vs untreated controls

Nasal epithelial brushings from 15 CF individuals (homozygous or compound heterozygous for the *F508del* mutation) were collected. Ciliated and goblet cells were observed in freshly isolated CF nasal cells (not shown). Isolated cells were expanded under conditional reprogramming culture method, that supports the long-term expansion of airway epithelial cells with the use of the Rho kinase (ROCK) inhibitor Y-27632. In a limited series of cases (*n* = 3) they were passaged until to obtain the fourth passage (Supplementary Fig. 1), showing a doubling time of 0.35 ± 0.08 days (mean ± SD), and evaluated for cytobiocompatibility with NiM formulations. The MTT assay evaluates mitochondrial metabolic activity in living cells and thus has been applied to infer cell viability. Results showed that NiM formulations were not cytotoxic versus control untreated cells in a 4-h incubation time frame, independently of ILO concentration (Fig. 3A). To assess whether longer times of incubation would be cytocompatible, hNECs were incubated for 24 h resulting in no cytotoxicity, except with NiM-PEG-ILO at 3500 nM (Fig. 3B).

Since toxicity of NiM formulations was more pronounced at 24 h, we estimated at this time point the IC50 of NiM-ILO and NiM-PEG-ILO by linear regression. The IC50 for NiM-ILO and NiM-PEG-ILO was 20,010 nM and 31,020 nM, respectively.

### Tightness and mucociliary differentiation

**Fig. 4:**
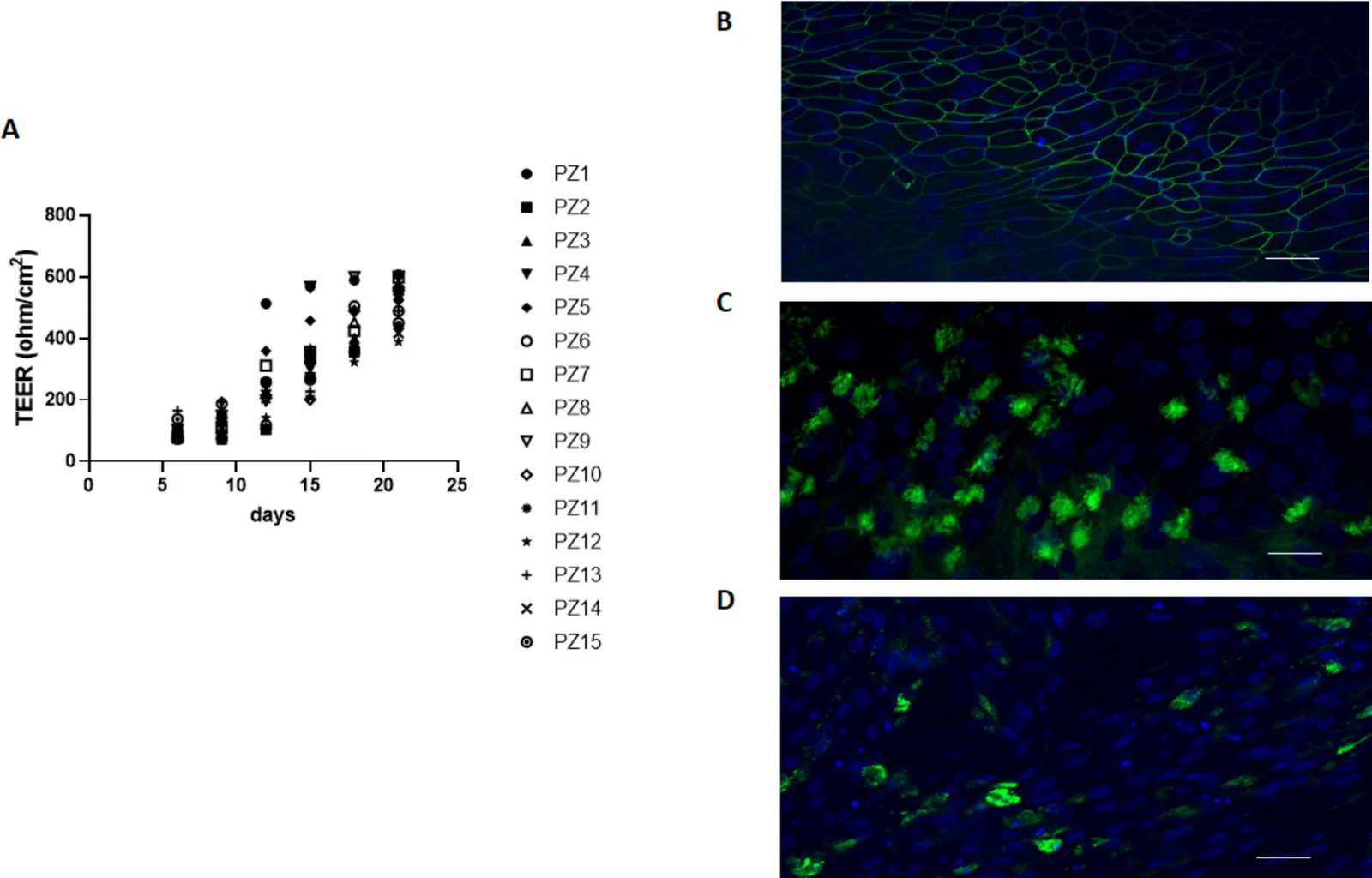
Tightness and mucociliary differentiation of CF hNECs. (**A**) Trans-epithelial electrical resistance (TEER) of hNECs at ALI. TEER was meaured on hNECs (*n* = 15) at the indicated times. (**B-D**). Representative images of immunofluorescence confocal microscopy of CF hNEC monolayer showing tight junctions (ZO-1) (**B**), cilia (acetylated-tubulin) (**C**) and mucin (MUC5AC) (**D**). Nuclei were labeled with DAPI (blue). Bar, 20 μm

Tightness of hNEC monolayers was assayed by the measure of trans-epithelial electrical resistance (TEER) during their culture in ALI conditions. CF primary cells developed a high TEER after a 21-day culture, indicating that a tight epithelium similar to the in vivo situation was obtained (Fig. 4A). Epithelial cells grown at ALI for 21 days were stained for the presence of tight junctions (ZO-1) and the presence of goblet (MUC5AC) and ciliated cells (acetyl-tubulin). A control sample incubated only with DAPI revealed no background green fluorescence (Supplementary Fig. 2). hNECs obtained from a CF patient bearing *F508del*/*G542X* mutations expressed ZO-1 in a typical chicken-wire pattern reflecting high TEER (Fig. 4B). Ciliated cells (Fig. 4C) and goblet cells (Fig. 4D) were readily detectable. Similar results were obtained with hNEC cultured from another CF patient carrying *F508del*/*N1303K* genotype. These data support that in our ALI conditions the mucociliary differentiation of primary hNECs obtained from CF patients occurred.

### The effect of NiM formulations on mRNA levels of pro-inflammatory cytokines

**Fig. 5:**
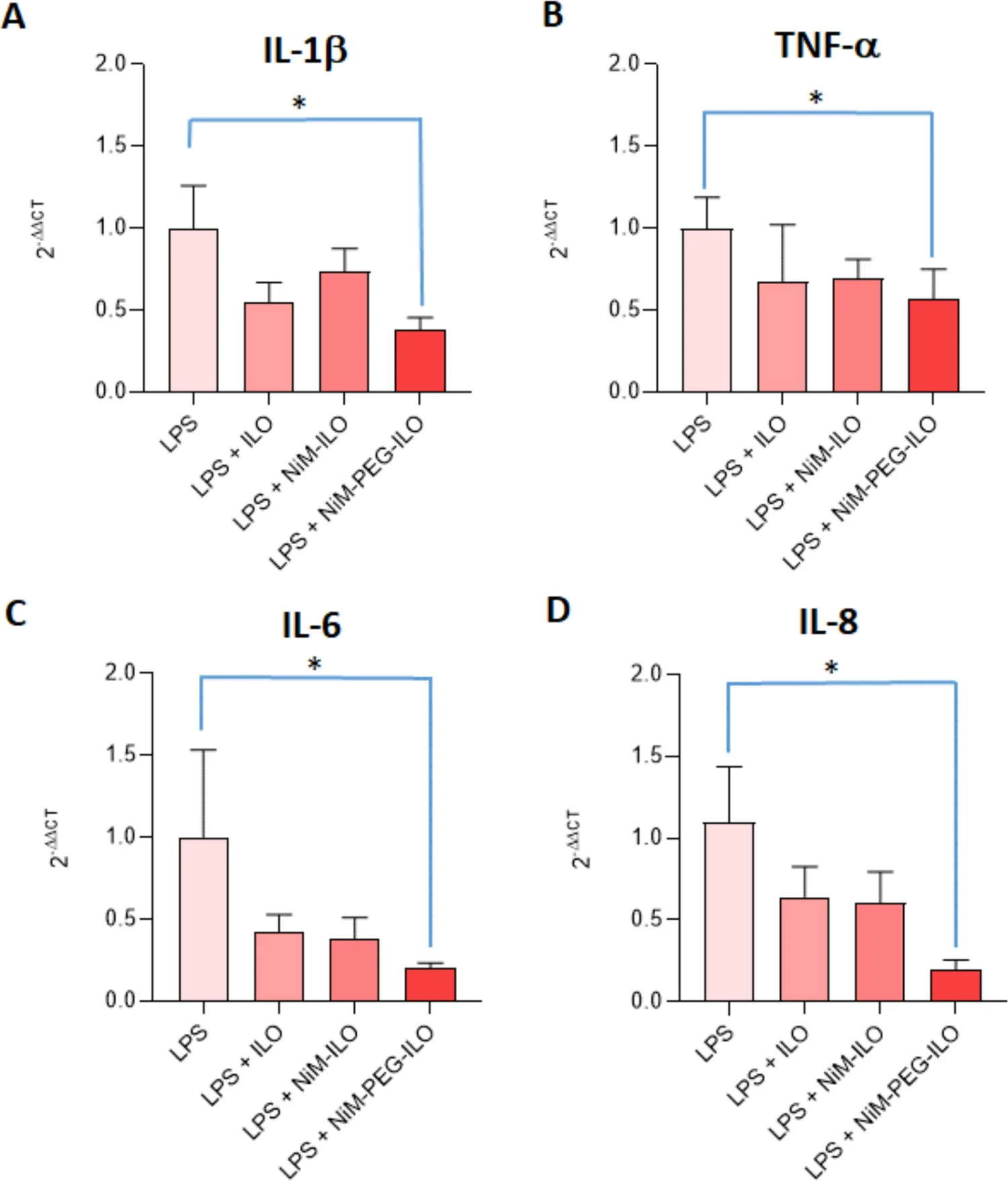
Effect of NiM formulations on cytokine gene expression. IL-1β (**A**), TNF-α (**B**), IL-6 (**C**) and IL-8 (**D**) mRNA levels were evaluated after hNECs were co-treated with LPS (10 μg/mL) and NiM-ILO or NiM-PEG-ILO (350 nM ILO) for 4 h. Bars show the mean expression values for each cytokine and error bars represent standard error of the means (*n* = 10 patients, each tested in duplicate). Statistical analysis: Kruskal–Wallis with Dunn’s multiple comparison test. *p < 0.05

After differentiation, hNECs at ALI were allowed to produce endogenous mucus for at least 4–5 days before challenges (i.e., apical medium was not changed within this time frame). To mimic in vivo conditions, LPS was added to the apical side in the absence or in the presence of free ILO or NiM formulations containing ILO-loaded pegylated and unpegylated nanoparticles (NiM-PEG-ILO and NiM-ILO samples, respectively) at drug concentration of 350 nM, based on the cytobiocompatibility assay. The concentration of LPS used in this study (i.e., 10 g/ml) was already used in CF and non-CF immortalised cell lines as grown on plastic [20, 32, 33] and as ALI cultures [34, 35] to study proficiently the inflammatory response in terms of cytokine secretion. Figure 5 reports data obtained after 4 h of incubation and assessment of cytokine mRNA levels. As shown, ILO, NiM-ILO and NiM-PEG-ILO reduced mRNA levels of all cytokines tested (IL-1β, TNF-α, IL-6, and IL-8). NiM-PEG-ILO was the most effective treatment, leading to a statistically significant decrease in all four cytokines compared to LPS alone.

### The effect of NiM formulations on miRNAs involved in inflammation

**Fig. 6:**
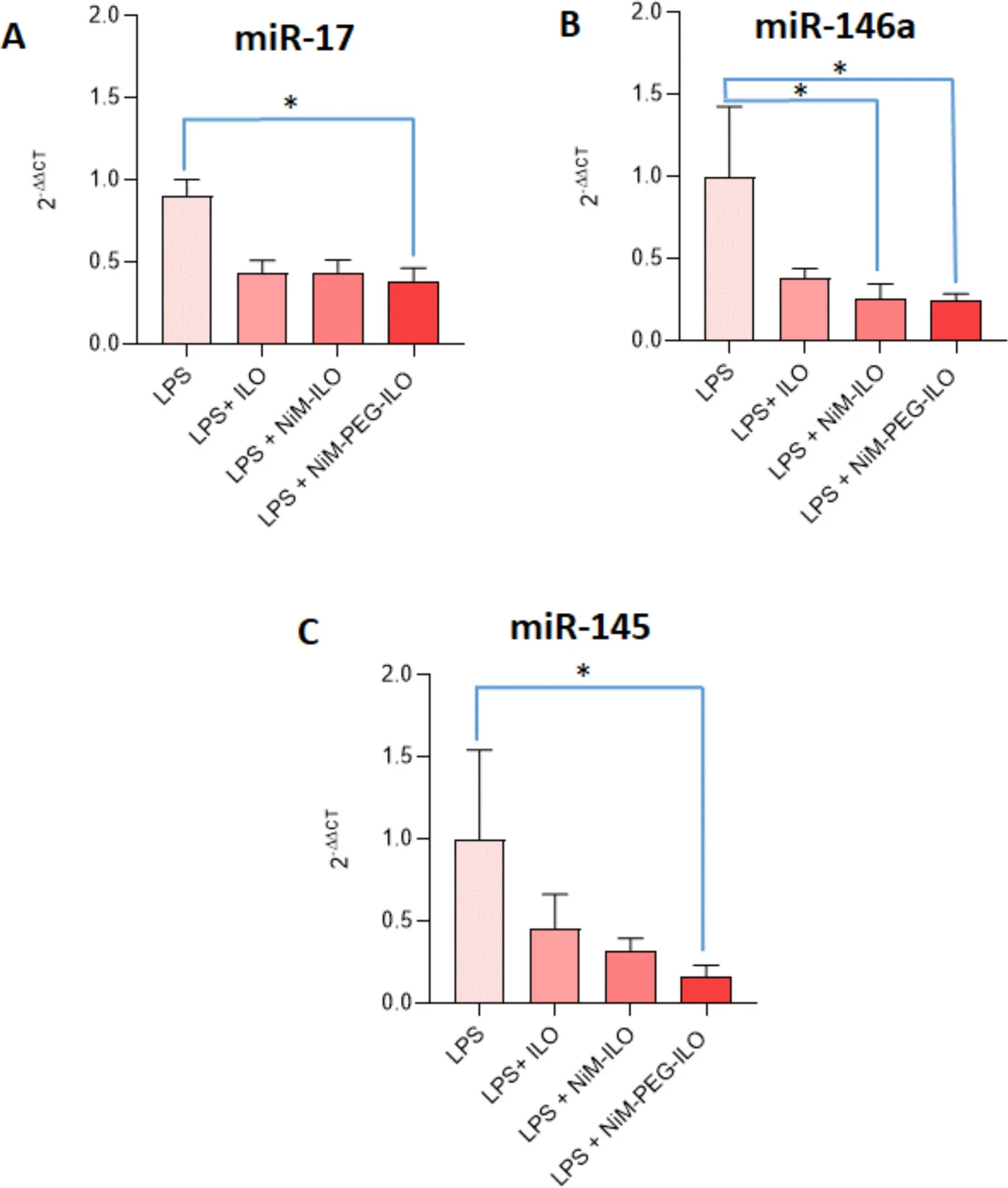
Effect of NiM formulations on miRNA expression levels. miR-17 (**A**), miR-146a (**B**) and miR-145 (**C**) levels were evaluated after hNECs were co-treated with LPS (10 μg/mL) and NiM-ILO or NiM-PEG-ILO (350 nM ILO) for 4 h. Bars indicate the mean expression levels of miR-17, miR-146a, and miR-145, and error bars represent the standard error of the means (*n* = 10 patients, each tested in duplicate). Statistical analysis was performed using the Kruskal–Wallis test. *p < 0.05

Under the experimental conditions outlined above, we evaluated the expression levels of those miRNAs involved in the regulation of pro-inflammatory cytokines IL-1β, TNF-α, IL-6 and IL-8. Based on the literature [4] and in silico analysis (see Materials and Methods), we selected miR-17, miR-146a, miR-145. Figure 6 shows that a reduction was observed for all miRNAs tested; however, only NiM-PEG-ILO treatment showed statistically significant effects, except for miR-146a, which was also significantly reduced by NiM-PEG.

### Correlation of inflammatory markers with clinical parameters

**Fig. 7:**
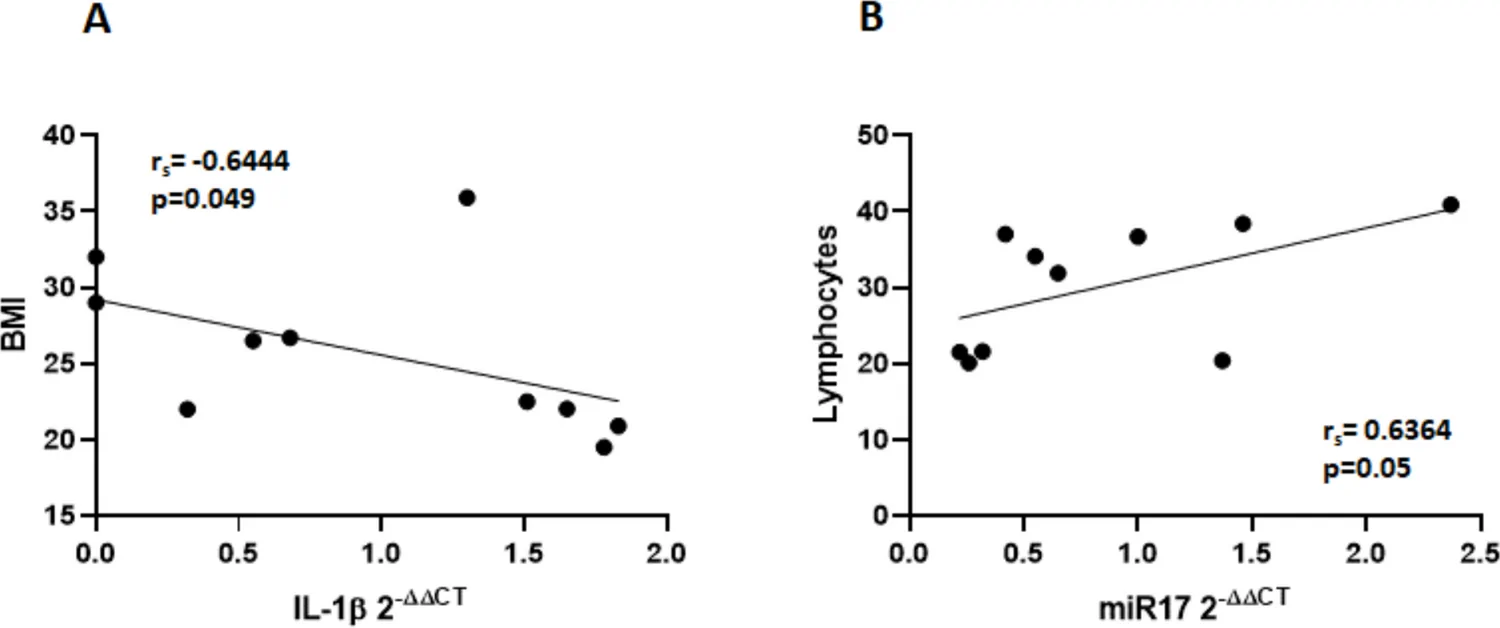
Correlation between inflammatory markers and clinical parameters. (**A**) Negative correlation between BMI and IL-1β. (**B**) Positive correlation between lympohocyte (% of total WBC) and miR-17

Next, we considered whether inflammatory markers (cytokines and miRNAs) evaluated in this study were correlated with clinical parameters (FEV1, BMI, CRP, WBC, and differential counts), considering 2^−ΔΔCT^ as basal levels in control untreated cells. There was a negative correlation between IL-1β levels and BMI (Fig. 7A), while a positive correlation between miR-17 levels and lymphocytes (Fig, 7B). Other noteworthy correlations are presented in Table 4. Interestingly, TNF-α correlated positively with monocytes and negatively with basophils, whereas IL-6 correlated positively with eosinophils. Of miRNAs, miR-17 correlated negatively with both CRP and neutrohils, while miR-145 was correlated negatively with basophils.

**Table 4:** Correlations between inflammatory markers and clinical parameters

	BMI	CRP	Neutrophils	Lymphocytes	Monocytes	Basophils	Eosinophils
IL-1β	r_s_ = −0.64 *p* = 0.049						
TNF-α					r_s_ = 0.55 *p* = 0.09	r_s_ = −0.60 *p* = 0.06	
IL-6							r_s_ = 0.05 *p* = 0.08
miR-17		r_s_ = −0.60 *p* = 0.07	r_s_ = −0.60 *p* = 0.07	r_s_ = 0.63 *p* = 0.05			
miR-145						r_s_ = −0.56 *p* = 0.09	

### Uptake of fluorescent NiMs

**Fig. 8:**
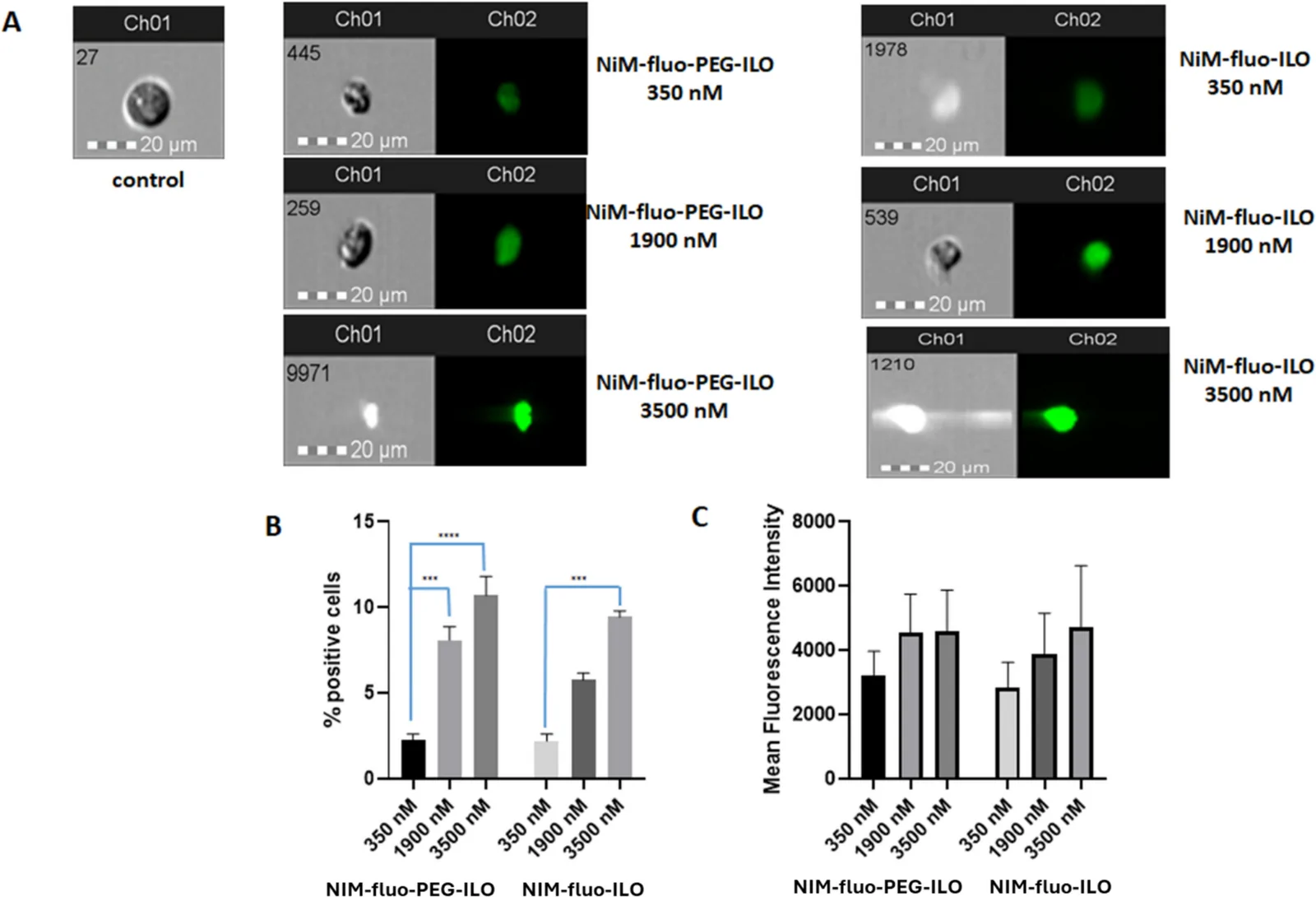
Uptake of fluorescent NiMs by differentiated hNECs. After incubation with NiMs for 4 h, cells were treated with trypan blue, detached and analysed by cytofluorimetry. **A** Brightfield and green fluorescent images of representative single cells treated in various conditions are shown. **B** and **C** show percentage of positive cells and mean fluorescent intensity, respectively. Data were obtained from the analysis of three patients’ hNECs. ***p = 0.0001; ****p < 0.0001

To understand whether these results were due to the internalization of the drug-loaded polymeric nanoparticles, we undertook an uptake assay by using NiMs formulations containing pegylated and unpegylated FITC-conjugated polymeric nanoparticles, NiM-fluo-PEG-ILO or NiM-fluo-ILO. Differentiated hNECs were analyzed after challenge with three different doses of either NiM-fluo-ILO or NiM-fluo-PEG-ILO. Cytofluorimetric analysis revealed the cell population that was gated for fluorescence evaluation after excluding dead cells and cells debris (Supplementary Fig. 3). Results, represented in Fig. 8, show that both formulations gained a dose-dependent increase in the uptake of nanoparticles and in mean fluorescence intensity. This is evidenced by fluorescent images (Fig. 8A) and by quantitative analysis (Fig. 8B and C). However, the percentages of positive cells were not different between the two formulations at all concentrations (Fig. 8B). Although the mean fluorescence intensity increased with doses (Fig. 8C), this increment was not significanly different among doses, indicating that NiMs gained entrance in more cells but not as regarding their amount.

## Discussion

To take advantage of the mucopenetrating properties of polymeric-based nanoparticles embedded in mannitol microparticles (Nano into Micro formulations, NiM), we have considered a novel approach to counteract the hyper-inflammatory state in CF airways. The hyper-inflammatory disease is considered the culprit of damage in the CF lungs, leading to bronchiectasis and pulmonary insufficiency. However, a precision medicine to hit potential targets is far to be accounted, likely also for the complexity of the inflammatory process, involving epithelial cells, monocyte-macrophages and polymorphonuclear cells (neutrophils) [36]. Taking into account the lack of precise targets in the CF hyperinflammatory lung disease, we undertook this study to understand in a cohort of CF patients (all > 18 years, homozygous or heterozygous for the *F508del* mutation, and with prior treatment with the triple therapy Trikafta) the anti-inflammatory effects of nanoformulations on primary nasal cells, used in a pre-clinical model for drug discovery and implementation [22]. The CRC method was used to culture nasal cells to expand the number of available hNECs, but differently to other studies [37], we did not use murine fibroblasts as feeder layer to avoid xenoproteins contamination. Rather, addition of the ROCKi Y-27632 to the primary HNECs quickly induces the adult stem cell phenotype, undifferentiated hNEC cell state and longer basal cells proliferation [38]. In our study, upon switching to ALI conditions, hNECs readily formed a tight epithelium with positive staining by using antibodies against ZO-1, a TJ protein, and MUC5AC and acetylated-tubulin, testifying that we are dealing with a truly mucociliary differentiated airway epithelium.

Epithelial cells, together with macrophages and neutrophils, are known to contribute to the expression and release of pro-inflammatory cytokines in the airways of patients with CF [39]. In particular, IL-1β, TNF-α, IL-6, and IL-8 are among the key mediators involved in the chronic inflammatory response. hNECs express Toll-like receptors (TLRs) on mRNA and protein level, including TLR-4, the receptor for LPS [40]. They also express myeloid differentiation primary response gene (MyD)88 [40], a transduction molecule involved in TLR-4 signaling, that leads to the activation of the nuclear factor-κB-dependent pathway to the cytokine secretion [41, 42]. At concentrations higher than that we used in this study, i.e. 10 µg/ml, LPS aggregates [43] and such large-size particles would interfere with the binding of LPS to TLR4, thus explaining the lack of LPS at doses higher then 10 µg/ml on the secretion of IL-8 [32]. In this study, all four cytokines were found to be significantly downregulated following treatment with NiM formulations containing Iloprost, confirming the anti-inflammatory potential of this strategy. These findings are particularly relevant in light of previous studies suggesting that certain cytokines, such as IL-8, are less responsive to pathological modulation in CF epithelial cells [44, 45]. However, in our preclinical model, IL-8 also showed a clear and significant reduction, indicating that, under appropriate conditions, CF airway epithelial cells can mount a coordinated response to treatment, effectively modulating a broad range of inflammatory mediators. This observation supports the hypothesis that NiM-based formulations may exert a systemic effect on the inflammatory microenvironment of CF airways. Further studies are warranted to assess whether this effect extends to other immune cell types involved in CF inflammation, such as macrophages and neutrophils.

MiRNA dysregulation has been implicated in CF pathophysiology. Some of these aberrantly expressed miRNAs have been demonstrated to be involved in the regulation of key components of inflammatory signalling and the unfolded protein response as well as in the regulation of the expression of CFTR itself [4, 46]. We monitored the effects of NiM formulations on miRNAs expression as a therapeutic strategy to modify the CF hyperinflammatory state [47]. In particular, miR-17 entails a complex role in CF. From studies involving bronchial brushings, βENaC-Tg mice and primary bronchial epithelial cells, Oglesby and colleagues [48] have proposed defective CFTR, inflammation, neutrophilia and mucus overproduction in regulation of miR-17. Further studies have to be pursued to understand whether the downregulation of miR-17 exerted by ILO-loaded nanoparticles embedded into the NiMs is beneficial for the CF airways in terms of the regulation of hyperinflammation. miR-146a upregulation is involved in CF inflammation. Indeed, the elevation of miR-146a causes macrophages isolated from CF patients to overproduce IL-6 in response to LPS stimulation [49]. MiR-145 is on the other hand involved in the remodeling and fibrosis processes caused by deregulation of TGF-β (transforming growth factor-beta) pathway. Particularly, miR-145 was found at increased levels in nasal airway cells of CF versus non-CF individuals and it correlated with a consistent decrease of SMAD3, a key component of the immune system which plays a protective effect against infection and inflammation [50]. Overall, NiM formulations can possibly modify the hyperinflammation condition of CF airways, however, their regulation of cyokines and miRNAs should be corroborated by miRNA modulation through miRNA mimics and antagomirs [51].

Nonetheless, some initial insights have emerged from correlations between cytokines and miRNA expression and clinical parameters. IL-1β levels correlated negatively with BMI, indicating this cytokine as a potential marker of malnutrition in CF patients. While IL-1β is associated with early structural lung damage in CF [52], BMI has not been found correlated to this cytokine. On the other hand, miR-17 levels correlated positively with circulating lymphocytes. Lymphocyte dysfunction in the CF lung disease pathogenesis and evolution has been invoked, since exaggerated Th17- and Th2-cell responses have been implicated in CF chronic lung inflammation [53]. Our data indicate that the role of miR-17 in this dysregulation should be further studied, however the therapy done with the triple combination of Trikafta could be reflected on this ex-vivo study. Finally, the sample size of our cohort study is small, although highly homogeneous from the genetical and clinical viewpoints, which should be improved in our subsequent studies. Nevertheless, these correlation studies allow a direct comparison between clinical and experimental outcomes, which are not possible on animals with CF [54], further strengthening the use of primary airway cells in ALI conditions and their use in pathophysiologic and drug screening and validation studies.

In a previous study [20], polymeric nanoparticles loaded with ILO, were successfully developed, exhibiting suitable physico-chemical characteristics and surface properties favorable for mucus diffusion. To enable their administration as a dry powder for inhalation, these colloidal carriers were effectively incorporated into mannitol-based microparticles. The resulting composite NiM particles displayed appropriate flowability and aerosolization performance, supporting its potential for direct pulmonary delivery in patients. The selection of mannitol as the matrix-forming excipient was based on its ability to rapidly dissolve upon contact with pulmonary fluids, enabling the prompt release of the embedded nanoparticles. Moreover, the osmotic effect generated by mannitol promotes water influx into the airway surface liquid, thereby facilitating nanoparticle diffusion through the thickened mucus characteristic of CF. Importantly, both NiM formulations were cytobiocompatible with hNECs and IC50 concentrations were much higher than those used in this study, which bodes well for an in vivo application.

The effects of ILO-loaded nanoparticles (released from the NiM formulations) on cytokine mRNAs and miRNAs follow their uptake by hNECs. This is possible since our ILO-loaded nanoparticles are designed for enhancing drug delivery to airway epithelial cells through rapid diffusion across the mucosal barrier. Once ILO-loaded nanoparticles take contact with hNECs they are readily internalized in a dose-depenent manner. However, no difference was noted between non-pegylated and pegylated formulations. We infer that the higher performance of pegylated formulations than those non-pegylated is to be attributed to the crossing of endogenous mucus overlying hNECs rather than to their uptake. However, further studies should focus on the time course and uptake mechanisms [55].

Limitations of this study include the lack of immune cells, i.e. macrophages and neutrohils, in our ex-vivo model. Macrophages and neutrophils participate to the deranged CF inflammatory response in the CF airways, and thus their presence would fully recapitulate the complexity of the in vivo situation. Further experiments, by adding immune cells either on the top of NECs in the upper chamber or in the lower chamber of Transwells, will be ncessary to undertake this task.

## Conclusion

The present study applies the novel Nano into Micro (NiM) particle as powdered formulation bearing a reporpused drug, Iloprost, to an ex-vivo model of airway epithelial cells, considered a proxy approach to the clinical application in cystic fibrosis. PEGylation was used as a strategy for giving nanoparticles stealth properties that enable them to overcome the endogenous mucus barrier. Inedeed, pegylated Iloprost-loaded polymeric nanoformulations embedded into inhalable microparticles could exert a downregulatory effect on pro-inflammatory cytokines and miRNAs in primary CF human nasal epithelial cells, paving the way to a more focused approach to precision medicine in CF anti-inflammatory therapy.

## Supplementary Information

Below is the link to the electronic supplementary material.

ESM 1 (DOCX 1.16 MB)

## Data Availability

All data relevant to the study are included in the article or uploaded as Supporting Information. All other relevant data are available from the lead contact upon reasonable request.
