# Supplementary material for: Iloprost-loaded inhalable Nano into Micro (NiM) formulations for treating hyper-inflammation in a pre-clinical model of cystic fibrosis airway epithelial cells
Source: Drug Deliv Transl Res. 2026 Mar 21;16(11):4276–92. doi: 10.1007/s13346-026-02101-0 (PMC13748055; doi:10.1007/s13346-026-02101-0)
Supplement: Supplementary file 1 [file 13346_2026_2101_MOESM1_ESM.docx]

**Iloprost-loaded inhalable Nano into Micro (NiM) formulations for treating hyper-inflammation in a pre-clinical model of cystic fibrosis airway epithelial cells**

Annalucia Carbone^1§^, Piera Soccio^2§^, Emanuela F. Craparo^3#^, Pamela Vitullo^4^, Sofia Bonsignore^3^, Pasquale Tondo^2,5^, Cinzia Scialabba^3^, Namra Sajid^1,6^, Sobhan Jahani,^1^ Salvatore Emanuele Drago^3^, Sante Di Gioia^1^, Donato Lacedonia^2,5^, Gennara Cavallaro^3^, Massimo Conese^1#^

^1^Department of Clinical and Experimental Medicine, University of Foggia, Foggia, Italy

^2^Department of Medical and Surgical Sciences, University of Foggia, Foggia, Italy

^3^Department of Biological, Chemical and Pharmaceutical Sciences and Technologies (STEBICEF), University of Palermo, Palermo, Italy

^4^Cystic Fibrosis Support Center, Ospedale “G. Tatarella”, Cerignola, Italy

^5^Institute of Respiratory Diseases, Policlinico Riuniti of Foggia, 71122 Foggia, Italy

^6^Department of Precision Medicine in Medical, Surgical and Critical Areas, University of Palermo, Palermo, Italy

**Supplementary Fig. 1** Morphology of hNECs at different passages. Representative images of hNEC captured at the isolation (A), after 2 passages (B) and after 4 passages (C). Photograohs were taken with a 20X objective.

**Supplementary Fig. 2** Immunofluorescence and confocal microscopy of control sample. hNEC were rown on Trasnwells for 21 days and stained only with DAPI. Bar, 20 μm.

**Supplementary Fig. 3** Gating strategy of NEC grown at ALI condition. The whole population of control (cells not incubated with NiMs) was gated (R2 in A) and evaluated for fluorescence intensity as BL1-A corresponding to FITC (B). In B, a marker separated negative cells (on the left) from positive cells (on the right). In the control, positive cells were set at 0.
